# Catalytic antibodies in the bone marrow and other organs of Th mice during spontaneous development of experimental autoimmune encephalomyelitis associated with cell differentiation

**DOI:** 10.1007/s11033-020-06117-8

**Published:** 2021-02-17

**Authors:** Kseniya S. Aulova, Andrey E. Urusov, Ludmila B. Toporkova, Sergey E. Sedykh, Yuliya A. Shevchenko, Valery P. Tereshchenko, Sergei V. Sennikov, Thomas Budde, Sven G. Meuth, Irina A. Orlovskaya, Georgy A. Nevinsky

**Affiliations:** 1 Institute of Chemical Biology and Fundamental Medicine, Siberian Branch of the Russian Academy of Sciences, Novosibirsk, Russia; 2 Institute of Clinical Immunology, Siberian Branch of the Russian Academy of Sciences, Novosibirsk, Russia; 3 Institut Für Physiologie I, Westfälische Wilhelms-Universität, Robert-Koch-Str. 27a, 48149 Munster, Germany; 4 Department of Neurology, Westfälische Wilhelms-Universität, Albert-Schweitzer-Campus 1, 48149 Munster, Germany

**Keywords:** Th mice, Development of experimental autoimmune encephalomyelitis, Hematopoietic stem cells differentiation, Lymphocyte proliferation in different organs, Catalytic antibodies

## Abstract

Exact mechanisms of autoimmune disease development are still yet unknown. However, it is known that the development of autoimmune diseases is associated with defects in the immune system, namely, the violation of the bone marrow hematopoietic stem cells (HSCs) differentiation profiles. Different characteristics of autoimmune reaction development in experimental autoimmune encephalomyelitis (EAE) prone Th mice characterizing T-lymphocytes response were analyzed using standard approaches. Profiles of several HSCs differentiation of bone marrow (BFU-E, CFU-E, CFU-GM, CFU-GEMM, T- and B-lymphocytes) of Th male and female mice during spontaneous development of EAE were noticeably different. Patterns of total lymphocytes, B- and T-cells proliferation in several different organs (bone marrow, blood, spleen, thymus, and lymph nodes) were also remarkably different. In addition, there were in time noticeable differences in their changes for some organs of male and female mice. Characters of changes in the profiles of CD4 and CD8 cells proliferation in some organs not always coincide with those for total T lymphocytes. The changes in the differentiation profiles of HSCs and the level of lymphocytes proliferation in the bone marrow and other organs were associated with the increase in the concentration of antibodies against DNA, myelin basic protein, and myelin oligodendrocyte glycoprotein, and catalytic antibodies hydrolyzing these substrates. Despite some differences in changes in the analyzed parameters, in general, the spontaneous development of EAE in male and female mice occurs to some extent in a comparable way.

## Introduction

Multiple sclerosis (MS) is pathology of the central nervous system (CNS) associated with the occurrence of increased numbers of macrophages and T lymphocytes. The precise cause of MS pathology remains unknown [1]. Many studies support the important role of autoimmune (AI) reactions in the destruction of myelin. The activated myelin-reactive CD4^+^ lymphocytes could be mediators of MS [1]. Several recent publications also validate the B cells and autoantibodies (auto-Abs) against myelin autoantigens’ important role in MS pathogenesis [1–3].

The increased amounts of auto-Abs and the accumulation of B cells in the cerebrospinal fluid (CSF), together with the typical lesions in MS patients, provide key evidence for the involvement of demyelination to the humoral response [4]. Studies of animal models speak in favor that auto-Abs to myelin components may be involved in Ab-dependent demyelination [3]. Auto-Abs to cell protein-oligodendrocyte progenitors can interfere with remyelination by impeding or removing these cells [5].

Autoimmune diseases (AIDs) have first been suggested may be originated from defects of hematopoietic stem cell (HSC) [6]. Later, it was demonstrated that the spontaneous and antigen-induced development of EAE in C57BL/6 mice [7–10], as well as systemic lupus erythematosus (SLE) in MRL-lpr/lpr mice [11–13], is beginning due to immune system-specific reorganization of bone marrow HSCs. Defects of immune system include specific changes in the differentiation profile of bone marrow HSCs in combination with the production of catalytic antibodies (abzymes) hydrolyzing DNA, polysaccharides, peptides, and proteins. The appearance of enzymatic activities of Abs is the earliest and most statistically significant marker of many AIDs in humans and mammals [7–24], including SLE [18–20], MS [14–24], and EAE [7–10] in AIDs of some prone experimental mice. Abzymes enzymatic activities are well detectable at the pre-disease stage before the appearance of typical known markers of different AIDs [7–13, 25–30]. Concentrations of auto-Abs against various antigens at the pre-disease stage and at the beginning of different AI diseases usually correspond to the range of indices, which are typical for healthy humans and experimental healthy mice. The appearance of abzymes may indicate for the beginning of AIDs when the increase in their activities is associated with the development of deep pathologies [7–10]. Taken together, the development of various AIDs may be conditioned by multiple mechanisms providing a self-tolerance breakdown.

There are several different EAE models mimicking a specific facet of human MS, including C57BL/6 mice (for a review see [31–33]). The development of EAE in C57BL/6 mice has a spontaneous chronic-progressive course. These mice are characterized by specific B and T lymphocytes responses to antigens [31–33]. C57BL/6 mice were used recently to analyze possible mechanisms of spontaneous, myelin oligodendrocyte glycoprotein (MOG)- and DNA-accelerated EAE development [7–10]. In addition, immunizing of mice with MOG or DNA results in an acceleration of EAE development, associated with specific changes in profiles of bone marrow HSC differentiation, lymphocyte proliferation, and the production of Abs hydrolyzing MBP, MOG, and DNA.

There is another Th model of spontaneous CNS autoimmunity. This model was generated using the crossing of myelin-specific T-cell receptor (TCR) transgenic mice and myelin-specific immunoglobulin heavy chain knock-in mice [34]. Th mice are characterized by T-cells response to antigens leading to spontaneous development of a severe form of EAE. To understand the EAE development, it is important to reveal important parallel and complementary mechanisms of pathology development.

Here we studied for the first time profiles of differentiation of BFU-E, CFU-E, CFU-GM, and CFU-GEMM cells, T- and B-lymphocytes of Th mice bone marrow during spontaneous development of EAE. Changes in proliferation profiles of total lymphocytes, B, T, CD4, and CD8 cells in different organs were also analyzed. The concentration of Abs against DNA, MBP, and MOG, as well as relative activities of IgGs hydrolyzing these substrates at different stages of EAE development in male and female mice, were compared.

## Materials and Methods

### Materials

Bovine polymeric DNA, proteins, the Superdex 200 h 10/30 column, and Protein G-Sepharose and other different chemicals were obtained from GE Healthcare (New York, USA) and Sigma-Aldrich (Munich, Germany). Human MBP of 18.5 kDa was perched from RCMDT (Moscow, Russia), while MOG_35–55_ was from EZBiolab (Germany). All preparations were free from oligosaccharides, lipids, nucleic acids, and other possible contaminants.

### Methods

#### Experimental animals

Th inbred mice (3 months of age) were grown in the mouse breeding facility of the Institute of Cytology and Genetics (ICG) in standard conditions free of any bacterial, viral, and other pathogens. All experiments were carried out pursuant to protocols of the Bioethical ICG Committee corresponding to humane principles of work with animals of the European Communities Council Directive 86/609/CEE. The Bioethical ICG committee supported our study.

#### ELISA of anti-proteins and anti-DNA antibodies

The levels of anti-DNA Abs (sera were diluted 100-fold) were determined using standard ELISA: plates with immobilized double-stranded DNA, horseradish peroxidase-conjugated mouse Abs against human IgG of the test system ORGENTEC Diagnostika (Germany) were used according to the manufacturer's instructions as in [7–13].

The relative content of anti-MBP and anti-MOG IgGs was estimated using purified mouse sera polyclonal electrophoretically homogeneous IgGs according to [7–13]. MBP or MOG (0.01 mg/ml) in sodium carbonate buffer (50 μl, pH 9.6) was added to ELISA strips, which were incubated overnight at 23 °C. The strips were washed with TBS buffer (20 mM Tris–HCl containing 0.15 M NaCl) supplemented with 0.01% NaN_3_ and 0.05% Triton X-100 and three times with the same buffer containing no Triton X-100. To block the strip surfaces, they were treated for 2.5 h at 30 °C using TBS containing 0.2% egg albumin and 0.01% NaN_3_. The strips were washed 8 times with water and then with TBS containing 0.01% NaN_3_. The strips were incubated with 100 μl of TBS containing 1 μg/ml conjugate of monoclonal anti-human IgGs with horseradish peroxidase for 40 min at 30 °C rewashed 10 times with water. After adding 60 μl citric-phosphate buffer containing 3,3′,5,5′-tetramethylbenzidine, and H_2_O_2_, the strips were incubated for 14 min at 23 °C, and the reaction was stopped by the addition of 60 μl of 50% H_2_SO_4_. The relative concentrations of anti-MBP and anti-MOG IgGs were expressed as an optical density of the solution at 450 nm (units A_450_; an average of 3 measurements), which was determined using a Uniskan II plate reader (MTX Lab Systems, USA). Final concentrations of anti-MOG and anti-MBP IgGs in the samples were expressed as a difference in the relative A_450_ values of experimental and control samples; controls were without MOG or MBP.

##### IgG purification

Electrophoretically homogeneous IgGs of mice were obtained using the first affinity chromatography of sera proteins on Protein G-Sepharose and then FPLC gel filtration in drastic conditions (pH 2.6) as described before [7–24]. To protect IgGs from viral and bacterial contamination IgGs, they were filtered using Millex membranes (0.1 μm) as described before [22–24]. SDS-PAGE of IgGs was performed using 4–15% gradient gels and visualized by silver staining [7–24].

### DNA-hydrolyzing activity assay

DNase activity of IgGs was analyzed as in [7–13, 17, 18]. The mixtures (20 μl) contained 20 mM Tris–HCl (pH 7.5), 20 mM NaCl, 5 mM MgCl_2_, 1 mM ethylenediaminetetraacetic acid (EDTA), 20 μg/ml supercoiled (sc) pBluescript, and 0.001–0.1 mg/ml of IgGs. They were incubated for 5–24 h at 37 °C. DNA hydrolysis products were analyzed by electrophoresis using 0.8% agarose gels. Photographs of gels stained with ethidium bromide were analyzed by Gel-Pro Analyzer v9.11. The relative catalytic activity (RA) was calculated using the difference between intact supercoiled DNA (scDNA) and its relaxed form, taking into account DNA distribution between these bands after scDNA incubation in the absence of IgGs. All initial reaction rates of DNA hydrolysis were analyzed using linear parts of the time dependencies (20–40% of scDNA hydrolysis) and concentrations of IgGs, providing 20–40% of DNA hydrolysis. A complete transition of scDNA to its hydrolyzed forms was taken for 100% of the activity. The RAs (% of the hydrolysis) were finally recalculated to the same standard time and IgGs concentration.

### Protease activity assay

The reaction mixtures (10–50 μl) contained 20 mM Tris–HCl buffer (pH 7.5), 0.7–1.0 mg/ml of proteins (MOG or MBP), and 0.001–0.2 mg/ml of IgGs as in [7–10, 14–16]. The mixtures were incubated for 5–24 h at 37 °C. The MOG or MBP cleavage products were analyzed by SDS-PAGE using 12% or 3–15% gradient gels and following Coomassie R250 staining. The gels were scanned, and products of their hydrolysis were quantified using GelPro v3.1 software. The RAs of various IgGs were evaluated from a percentage decrease in the initial proteins transited to their hydrolyzed forms. The hydrolysis of MOG or MBP incubated without Abs was taken into account. All initial rates of MOG or MBP hydrolysis were estimated using condition of the pseudo-first-order reaction considering linear regions of time dependencies and concentrations of IgGs (20–40% hydrolysis of the proteins).

### SDS-PAGE analysis of catalytic activities

Analysis of DNA-, MBP-, and MOG hydrolyzing activities of Th mice IgGs after SDS-PAGE was performed as described in [14–23]. IgGs were pre-incubated at 25 °C for 30 min under nonreducing conditions (50 mM Tris–HCl, pH 7.5, 1% SDS, and 10% glycerol). After SDS-PAGE of IgGs to restore the catalytic activities of IgGs, SDS was taken away by the gel incubation for 1 h at 20 °C with 4 M urea and then washed 10 times (7–10 min) with H_2_O. Then the gel 2–4-mm cross-sections of longitudinal slices were cut up thoroughly destroyed and incubated with 50 mM Tris–HCl buffer, pH 7.5, containing 50 mM NaCl 5 (50 μl) for 5–7 days at 4 °C to allow proteins refolding and eluting from the gel. The gel was separated by centrifugation and solution obtained used for assay of DNase and protease activities, as described above. Parallel control longitudinal lanes were used for detecting the position of IgGs on the gel by silver staining.

### In culture analysis of bone marrow progenitor cells

Bone marrow samples from mouse femurs were obtained, and the bone marrow cells’ ability to form colonies was estimated as in [7–13]. Four dishes per one mouse (2 × 10^4^ cells) were grown using the specific for mouse cells standard methylcellulose-based M3434 medium (StemCell Technologies, Canada). The medium contained stem cell factor, erythropoietin (EPO), interleukins IL-3, and IL-6. The relative number of colonies of CFU-GM, CFU-GEMM, BFU-E, CFU-E cells was calculated on the dishes after 14 days of the incubation at 37 °C (5% CO_2_) in a humidified incubator as in [7–13].

### Evaluation of lymphocytes in samples of different mouse tissues

The relative content of B and T lymphocytes in the blood and different organs of mice was determined using flow cytometry. Peripheral blood was obtained using standard decapitation of mice. Sodium citrate was used as an anticoagulant. After cell counting, 500 thousand leukocytes (but not more than 150 μl) were taken for cytometric analysis. Cells were incubated with monoclonal antibodies in the dark for 20 min, then red blood cells in blood samples were lysed for 20 min using a tenfold volume of RBCLysisBuffer lysis buffer (Biolegend, San Diego, CA, USA). Then, the cell samples were centrifuged for 10 min and washed by centrifugation with 500 μl of PBS containing 0.02% EDTA and 1% sodium azide. After centrifugation, 50 μl of PBS was added to the cell pellet and analyzed on a flow cytometer.

Lymphocytes were isolated from blood, bone marrow, thymus, lymph nodes, and spleen. Bone marrow was gained by washing the cavity of the femur. Lymph nodes and thymus were carefully homogenized in a glass homogenizer, large particles were removed, and cells were resuspended by passing a disposable syringe through a needle. Spleen cells were obtained by washing the organ with a syringe with a medium through punctures in the stroma of the spleen. This method allows us to get splenocytes without impurities of the organ stroma. Cells were washed 2 times by centrifugation RPMI-1640 medium (5 ml) for 10 min at 1500 rpm. After the second centrifugation, 1 ml of RPMI-1640 medium containing 10 mM HEPES, 10% fetal bovine serum (Invitrogen, Waltham, MA, USA), 0.5 mM 2-mercaptoethanol, 2 mM l-glutamine, 100 μg/ml benzylpenicillin, and 80 μg/ml gentamicin was added to the cell pellet and the cells were counted. To analyze the relative cell content in extracts of various organs, 500 thousand cells were used in 100 μl of PBS buffer containing 10% fetal bovine serum and the conjugates of different specific monoclonal antibodies. To analyze relative number of various cells, specific antiCD45-BV510 (Biolegendcat # 103138), antiCD3-FITC (Biolegendcat # 100204), antiCD4-PerCP (Biolegendcat # 100432), antiCD8alpha-APC (Biolegendcat # 126614), and antiCD19-PE (Biolegendcat # 115508) antibodies were used. All staining was carried out in accordance with the manufacturer's recommendations. Cells were incubated for 20 min with monoclonal Abs; they were washed by centrifugation after adding 500 μl of PBS containing 0.02% EDTA and 1% sodium azide. After centrifugation, 50 μl of PBS was added to the cell pellet and the mixture used for analysis by BD FacsVerse flow cytometer (BD Biosciences, SanJose, CA, USA). At least 100,000 events were collected for each sample. Gating was carried out as follows: the total population of lymphocytes was isolated in accordance with the size and granularity of these cells, and then the leukocyte population was determined using a pan-leukocyte marker CD45+, in which populations of CD3+ and CD3− leukocytes were isolated. In the CD3+ leukocyte population (T cells), CD4+ and CD8+ T cells were determined, and in the CD3− leukocyte population, the content of CD19+ B cells was estimated. For each group, the percentage ratio was determined relative to the initial lymphocyte population.

### Statistical analysis

The final values are given as the mean ± SD of at least three independent experiments for each mouse, averaged over 7 different male and female mice.

## Results

### Choosing a model for studying the mechanism of EAE development

T cell, according to literature, plays a leading role in the pathogenesis of human MS, while the B lymphocytes are also important for the development of this disease [1]. B cells provide the components of the humoral immunity of the adaptive immune system due to the secreting of Abs [35]. Mature B cells in the bone marrow have membrane receptors allowing them to bind different antigens against which they initiate an Abs response. Spontaneous and MOG-induced EAE in C57BL/6 mice characterizing by T and B cell responses are often used as a model of human MS [31–33]. It was shown that spontaneous development of EAE by C57BL/6 mice leads to slow changes during 2–3 months in the HSCs differentiation profiles, and the levels of lymphocyte proliferation in various organs were associated with the production of antibodies against MOG, MBP, and DNA [7–10]. Immunization of C57BL/6 mice with MOG results in a strong acceleration of EAE development with the appearance of the acute phase at 14–20 days after immunization [7–10]. During the onset of the disease (7–6 days) and the acute phase, a very strong change in the profile of HSC differentiation, an increase in the level of proliferation of sum of B and T lymphocytes, synthesis of antibodies against DNA, MBP and MOG, and abzymes hydrolyzing these substrates are observed. It should be noted that abzymes hydrolyzing DNA, MBP, and MOG are very dangerous for mammals. It was shown that abzymes with DNase activity penetrate through the cell and nuclear membranes, hydrolyze DNA of chromatin and stimulate cell apoptosis [36–38]. This leads to the increase in the blood concentration of DNA-histones complexes, which known as the main antigens of mammals leading to the production of Abs against DNA and histones [36–40]. Antibodies hydrolyzing MBP and MOG cleave these components in the composition of the membranes of nerve tissues, which leads to disruption of the nerve impulses [14–16].

As noted above, EAE prone C57BL/6 mice have both T- and B-cell responses of the immune system [7–10]. At the same time, Th mice also predisposed to the spontaneous development of EAE are characterized by a T-cell response [34]. Therefore, it was interesting to perform a detailed analysis of the changes in various parameters characterizing the spontaneous development of EAE in the case of Th mice. Previously, we analyzed the development of EAE in C57BL/6, using only male mice. MS is more common for women than for men [41]. Only a third of patients are male, although, in men, the disease often proceeds more severely and, in some forms, is less amenable to therapy. In addition, the disease occurs in men usually quite late. Taking this into account, in this study, a comparison of the development of MS in male and female Th mice was carried out.

### Hematopoietic progenitor colony formation

**Fig. 1:**
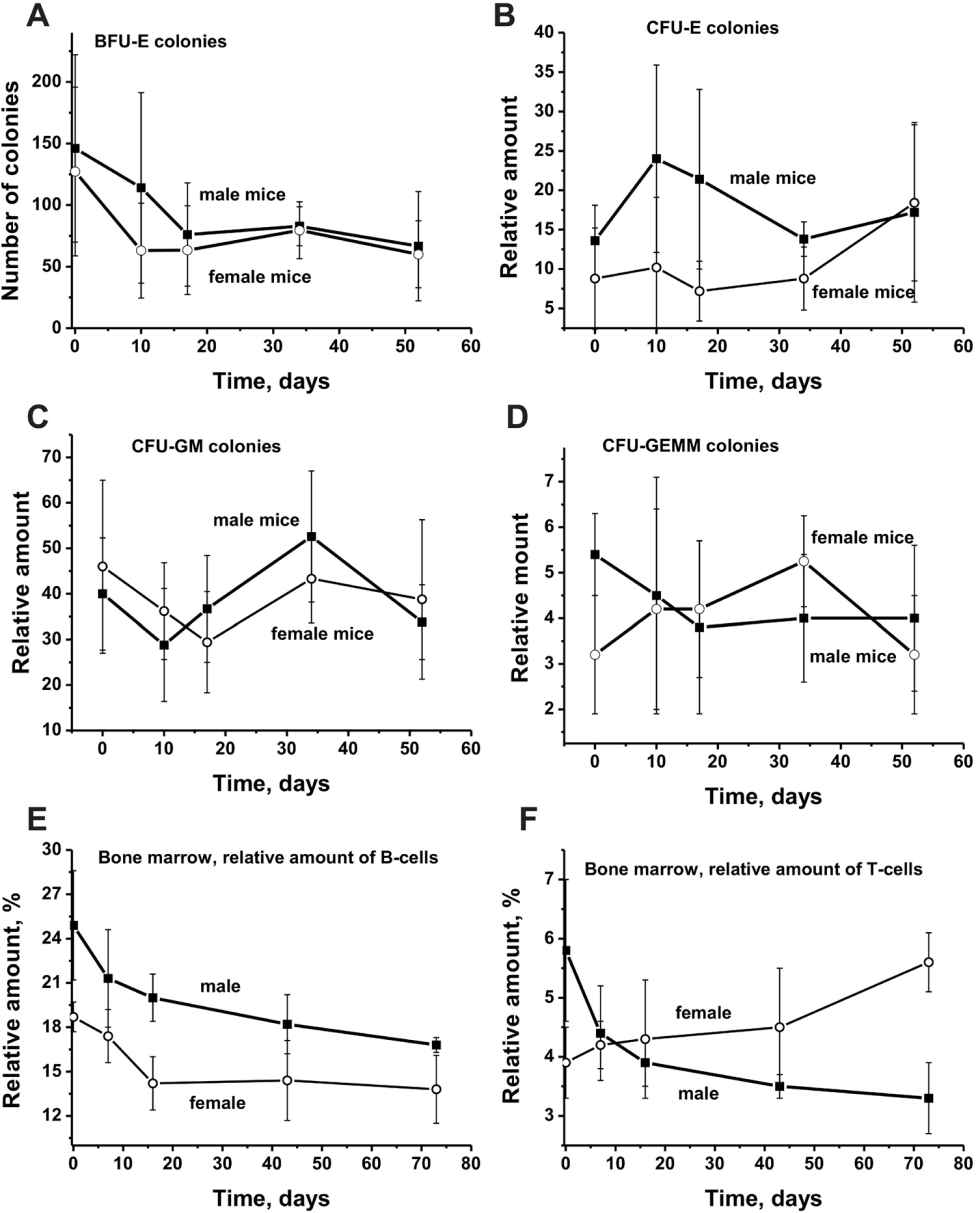
Changes over time in a number of Th male and female mice bone marrow BFU-E (**a**), CFU-E (**b**), CFU-GM (**c**), and CFU-GEMM (**d**) colony units. The number of all colonies is calculated for 15,000 bone marrow cells. Dependencies for male and female mice are shown in Panels

Spontaneous development of EAE in C57BL/6 mice results in significant changes in the differentiation profile of stem cells of bone marrow. Therefore, we first carried out a parallel analysis of the changes in the profile of the stem cells differentiation in three-month-old Th mice (7 males and 7 females), also predisposed to the development of EAE. Figure 1a shows that in male and female mice during 53 days of spontaneous EAE development in the bone marrow occurs a comparable 2.1 to 2.2-fold decrease in the relative number of BFU-E colonies (erythroid burst-forming unit, early erythroid forming unit). However, there was observed a significant difference in the change in the CFU-E colonies (erythroid burst-forming unit, late erythroid forming unit) in male and female mice (Fig. 1b). The average number of these colonies in males first increases to 10 days, and then it slowly decreases. In females, a slight change in the relative number of these colonies is observed up to about 35 days, and then their number begins to increase. Figure 1c demonstrates that the character of the change in the CFU-GM colonies (granulocyte–macrophage colony-forming unit) in male and female mice is complex but very similar. First, there is a decrease in the number of colonies at 10–20 days, then a notable increase by 35 days and again a perceptible decrease. Only in the case of CFU-GEMM colonies (granulocyte–erythroid–megakaryocytic–macrophage colony-forming unit), there is an opposite character in the change in the average number of CFU-GEMM colonies for male and female mice: the increase in the number of these clones in females and a decrease in males (Fig. 1d).

It is interesting that the total relative number of B and T cells in the bone marrow of male and female mice at the zero time of the experiment is significantly different (Fig. 1e and f). At the same time, in male and female mice, approximately the same 1.4- to 1.5-fold decrease in the relative number of B cells occurs. Absolutely unexpectedly completely opposite change in the number of T cells in the bone marrow of female and male mice; with time, the number of these cells in females increases, while in males, on the contrary, decreases (Fig. 1f). Thus, the differentiation profiles of bone marrow stem cells in male and female mice during the spontaneous development of EAE have significant differences.

The change in time in male and female mice of three types of cells (BFU-E, CFU-GM, and B cells) to some extent similar, but they differ for CFU-E, CFU-GEMM, and T cells (Fig. 1).

#### The relative content of B and T lymphocytes in various organs of mice

In the beginning, we estimated relative average values characterizing the content of total B, T, as well as CD4 and CD8 in various organs of male and female mice at 3 months of age (Table 1). Interestingly, the average number of B cells in different organs of mice was significantly different and decreased in order: spleen > blood ≈ thymus > bone marrow ≈ lymph nodes (Table 1). The relative content of T cells in different organs corresponds to another order in comparison to B lymphocytes: lymph nodes > spleen > blood ≈ thymus > bone marrow. In total, taking into account typical error in the determining the values, the content of B cells in different organs of male and female mice (Table 1) did not noticeably differ except bone marrow; in male mice, the content was ~ 1.3-fold (*P* < 0.05) higher than that of female mice. In addition, the content of CD4 and CD8 lymphocytes in the bone marrow of male was 1.5 to 1.8-fold higher (*P* < 0.05) than that of female mice. Thus, the spontaneous development of EAE in Th mice can lead to a change of relative amounts of B and T lymphocytes in different organs with slight differences for male and female mice.

**Table 1:** The average percentage content of different cells in various organs of male and female mice in three months of their life^a^

Organ	The relative content of different cells in various organs of mice, %				
	Sex	Total B cells	Total T cells	CD4 cells	CD8 cells
Bone marrow	Male	24.9 ± 1.5	5.8 ± 1.2	2.4 ± 0.6	2.4 ± 0.4
	Female	18.7 ± 1.0	3.9 ± 0.6	1.6 ± 0.3	1.3 ± 0.2
Blood	Male	32.0 ± 4.0	11.8 ± 2.2	6.4 ± 1.5	5.0 ± 1.6
	Female	33.1 ± 4.0	13.7 ± 3.2	7.1 ± 2.2	7.1 ± 1.5
Thymus	Male	32.0 ± 3.7	12.9 ± 0.5	7.7 ± 0.2	2.3 ± 0.02
	Female	32.0 ± 3.5	11.6 ± 1.3	6.6 ± 1.0	1.8 ± 0.4
Spleen	Male	63.1 ± 2.4	29 ± 1.7	19.7 ± 0.6	8.3 ± 1.2
	Female	59.8 ± 3.0	32.2 ± 2.5	20.3 ± 4.6	10.5 ± 2.7
Lymph nodes	Male	23.1 ± 3.3	65.2 ± 3.7	34.4 ± 3.8	24.4 ± 3.7
	Female	25.0 ± 3.7	69.9 ± 3.5	32.2 ± 3.5	27.1 ± 3.1

Each group of male and female mice contained five mice

^a^For each mouse in each group, three independent measurements were performed, and the average mean ± SD for five mice are given

**Fig. 2:**
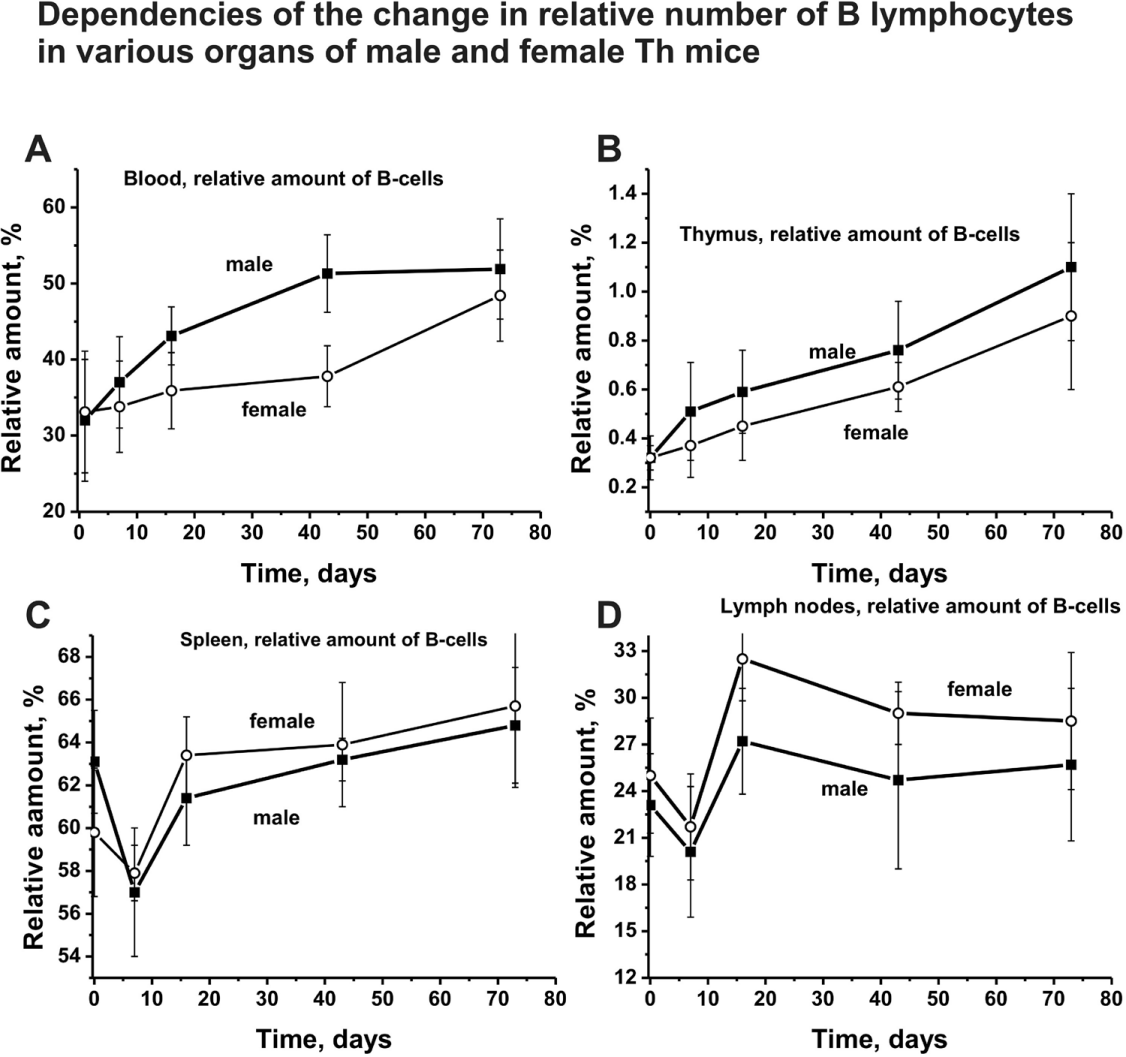
Over time dependencies in the relative number of B lymphocytes in different organs of Th male and female mice. Different organs and dependencies corresponding to male and female mice are shown in Panels

The changes in the relative amount of B lymphocytes in the bone marrow are shown in Fig. 1e, while in other organs of mice in Fig. 2. The relative number of B lymphocytes in the blood of male mice increased significantly faster over time than in female mice (Fig. 2a). The growth in the number of B lymphocytes in the thymus of male and female mice proceeded almost smoothly and similarly (Fig. 2b). The complex patterns of the changes in the average number of B lymphocytes in the spleen and of male and female mice were similar. First, during ~ 10 days of experiments, there was an extraordinary decrease in the number of B lymphocytes in spleen and lymph nodes, and then a remarkable rise at ~ 20 days was observed. Thus, on the whole, the dependencies of the changes in the relative concentration of B lymphocytes in different organs were different, but the character of these changes for each organ of female and male mice was, to some extent, the same (Figs. 1e and 2).

### The relative content of T lymphocytes in various organs of mice

**Fig. 3:**
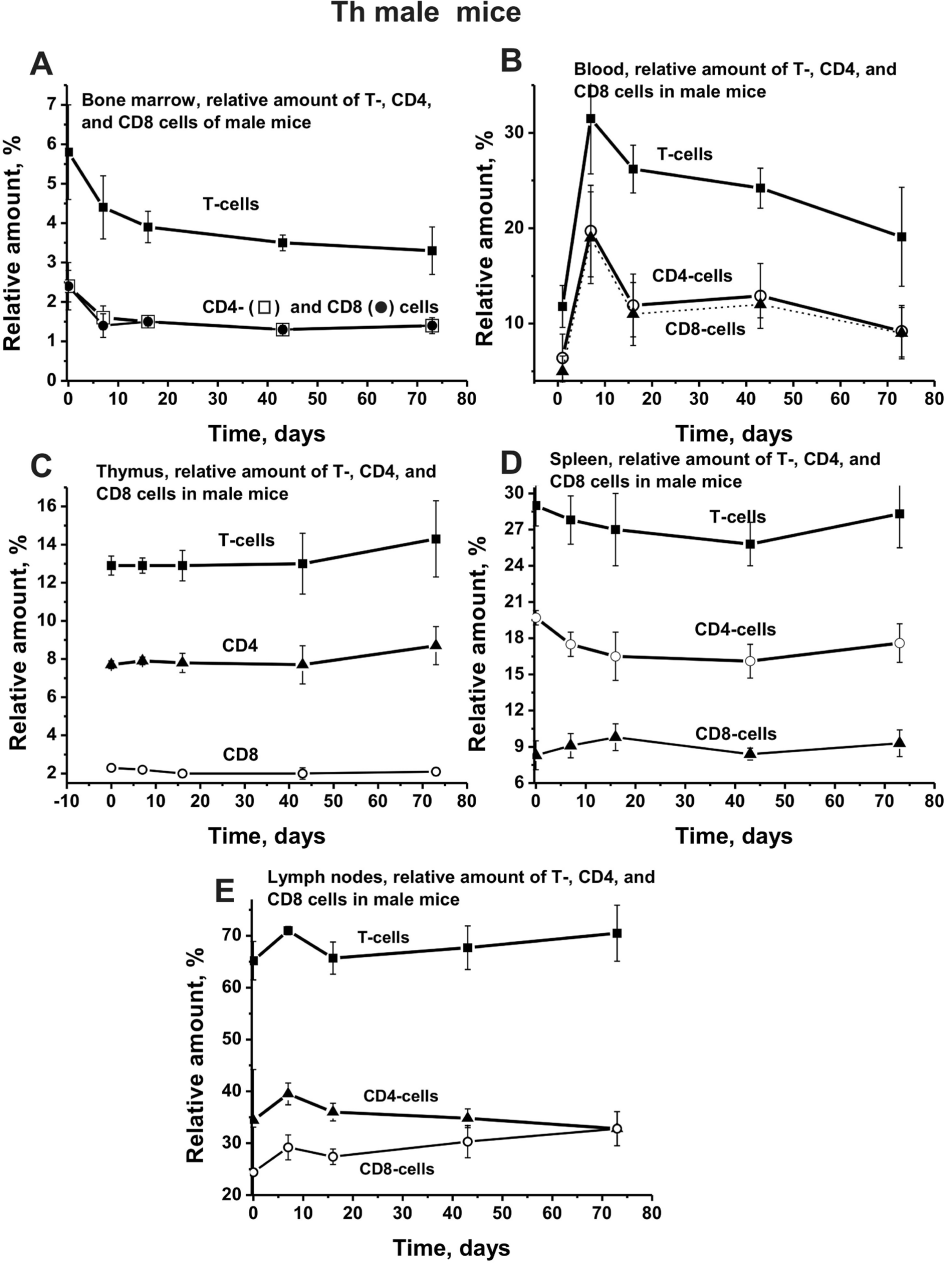
Over time dependencies in a relative number of total T, CD4, and CD8 lymphocyte in different Th male mice. Different organs and dependencies corresponding to male mice are marked in five Panels (**a**–**e**)

As mentioned above, patterns of changes in the relative number of T lymphocytes in the bone marrow of males and females are directly opposite (Fig. 1f). We have evaluated the time dependencies in various organs of mice the changes not only of the total number of T cells but also of CD4 and CD8 lymphocytes. A smooth decrease in time of the relative number of total T cells in the bone marrow of male mice correlated very well with the same kind of decrease in the number of CD4 and CD8 cells; the decrease curves for CD4 and CB8 lymphocytes are almost coincided (Fig. 3a). The character of the change in the total number of T cells in the blood of male mice was quite complex, with a marked increase in their number at ~ 10 days with their followed strong decrease (Fig. 3b). Quite a similar complex and almost matching dependencies were observed for change in the relative amount of CD4 and CB8 lymphocytes (Fig. 3b). While the number of CD4 and CD8 lymphocytes at zero time in the bone marrow and blood of male mice was almost the same (coinciding curves; Fig. 3a, b), the number of CD4 in the thymus was ~ 3.5-fold greater than that for CD8 cells (Fig. 3c). Interestingly, in the male thymus, there were no remarkable changes in the relative amount of total T lymphocytes, as well as CD4 and CB8 cells (Fig. 3c). A nearly similar situation was observed for CD4 and CD8 lymphocytes in the spleen of male mice (Fig. 3d). The number of CD4 was ~ twofold higher than that for CD8 cells, and similar to total T cells, there was no noticeable change in the relative amount of these lymphocytes in time (Fig. 3d). A slightly different situation was observed for T lymphocytes in the lymph nodes of male mice. At zero time, the relative number of CD4 was ~ 1.4-fold higher than that for CD8 cells. First, the increase in the number of total T lymphocytes, CD4, and CD8 cells by 10 days was observed, and then decrease in their number. However, after 16 days, the average number of CD4 cells was decreased, while CD8 lymphocytes, on the contrary, was constantly slightly increased (Fig. 3e). Thus, the dependencies of changes in the average relative number of lymphocytes in organs of male mice were remarkably different (Fig. 3).

**Fig. 4:**
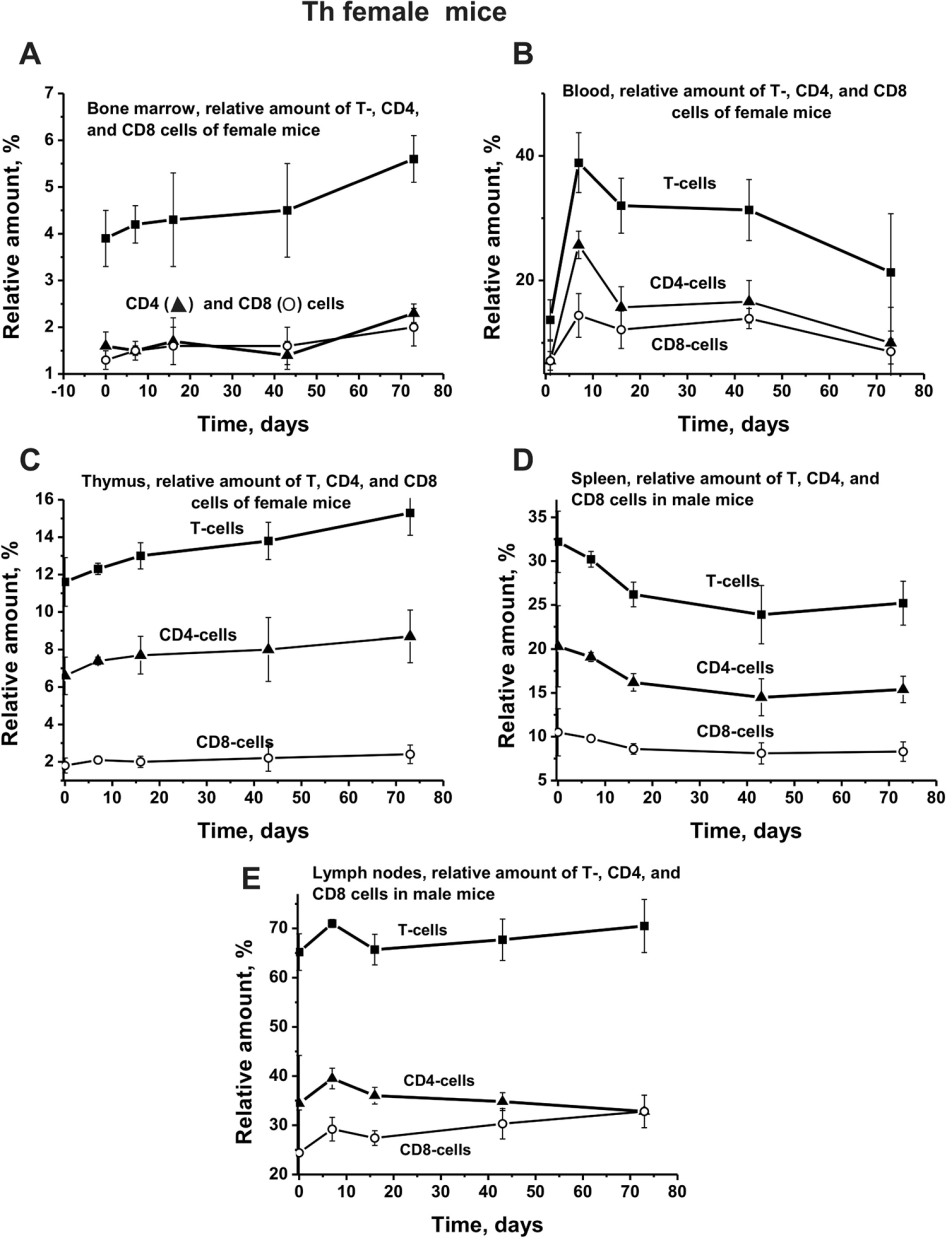
Over time changes in the number of total T lymphocyte, CD4, and CD8 cells in different organs of Th female mice. Different organs and dependencies corresponding to male mice are marked in five Panels (**a**–**e**)

In contrast to the bone marrow of male mice, the average number of T lymphocytes in the bone marrow of female mice did not decrease but increased in time (Fig. 1f). The relative amount of CD4 and CD8 in the bone marrow of female mice almost did not differ in time; the type of their change was the same as that for total T cells (Fig. 4a). The type of changes in T, CD4, and CD8 lymphocytes in female mice blood (Fig. 4b) was similar to that for male mice (Fig. 3b). The character of changes in time of the relative number of total T-, CD4, and CD8 cells in the thymus and spleen of female mice (Fig. 4c, d) was also comparable to those for male mice (Fig. 3c, d). At the same time, the patterns of changes in total T-, CD4, and CD8 cells in the lymph nodes of female mice (Fig. 4e) were, to some extent, different from those for male mice (Fig. 3e). When in male mice at about 10 days, an increase in the relative number of these cells was observed in females; on the contrary, their number was decreased markedly (Figs. 3 and 4). However, after 20 days, these dependencies for males and females become to be, to some extent, similar.

### Criteria analysis of catalytic activities of antibodies

**Fig. 5:**
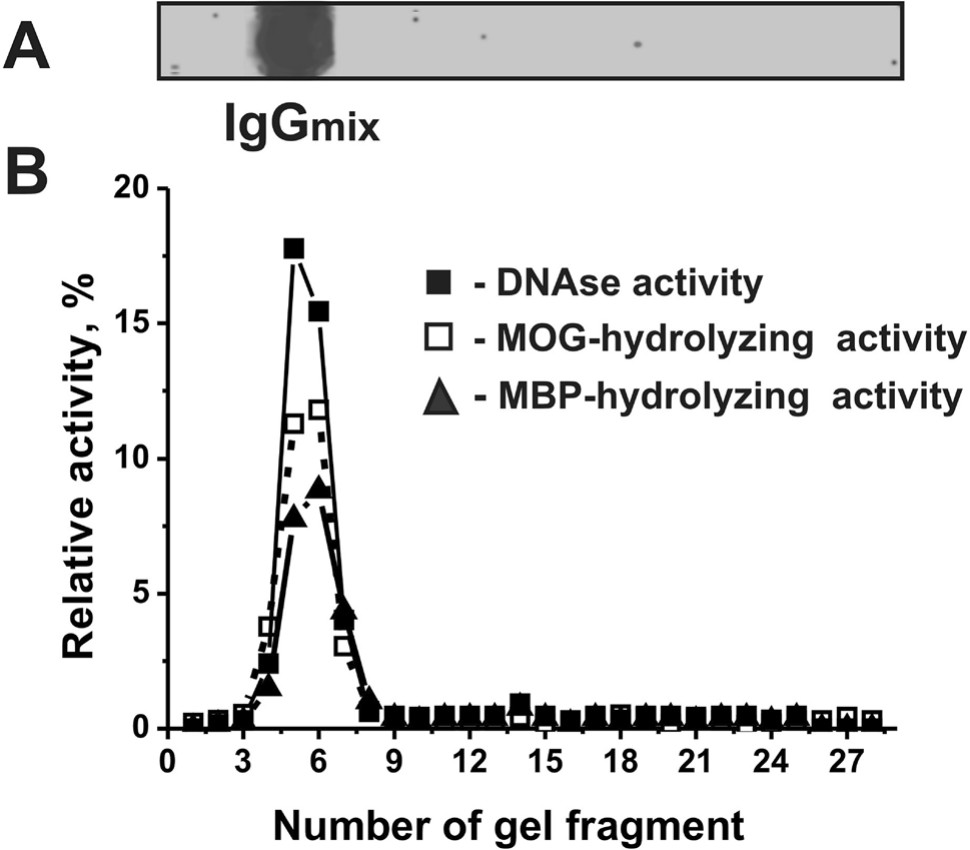
The homogeneity analysis by SDS-PAGE of 12 μg IgG_mix_ under non-reducing conditions (**a**); silver staining. The relative activities (RA, %) in the hydrolysis of DNA (filled square), MOG (open square), and MBP (filled triangle) by eluates of gel fragments were estimated using the extracts of gel fragments (2–3 mm) (**b**). Complete hydrolysis of these substrates after their incubation with eluates for 24 h of was taken for 100% (**a**). The errors of the RAs determinations from two independent experiments did not exceed 7–10%. **b** Position of IgGs

Here, we obtained electrophoretically homogeneous IgGs from sera of individual Th mice by chromatography of their proteins on Protein G-Sepharose in conditions removing nonspecifically bound proteins as in [7–10]. Then purified IgGs were additionally subjected for FPLC gel filtration. The homogeneity of IgGs was shown by SDS-PAGE with silver staining using equal amounts of Abs from sera of Th mice (IgG_mix_). (Fig. 5a). Similar to [7–10], we have used very strict criteria to show that DNA-, MOG-, and MBP-hydrolyzing activities are own properties of IgG_mix_ and are not due to co-purified canonical enzymes. It was shown that after SDS-PAGE, the positions of DNA-, MOG-, and MBP-hydrolyzing activities correspond to gel fragment containing intact IgGs, and there were no other protein bands (Fig. 5a) or peaks of catalytic activities (Fig. 5b).

### The relative content of Abs against proteins and DNA

**Fig. 6:**
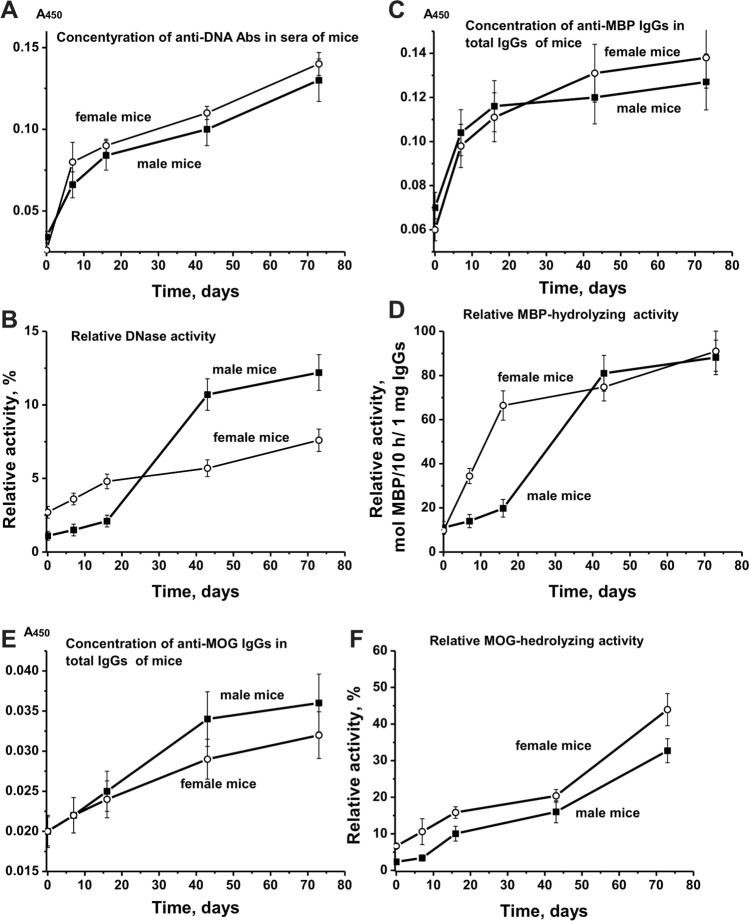
Over time changes in the relative concentration of Abs against DNA, MBP, and MOG and activities of IgGs in the hydrolysis of these substrates. Dependencies corresponding to concentrations and activities of male and female mice are marked in the Panels

The sera of healthy humans, as well as various animals, usually contain auto-Abs against DNA and different proteins in low concentration [25–30]. The average concentrations of Abs against DNA in sera of non-autoimmune BALB and CBA mice (at 2–15 months of age) as well as healthy SLE prone MRL-lpr/lpr mice (at 2–3 months of age) are usually low and varied from 0.03 to 0.04 A_450_ units [7–13]. The relative concentration of anti-DNA Abs (3 months of age) in sera of EAE prone C57BL/6 mice is higher (~ 0.11 A_450_ units), and it increased slowly during spontaneous development of pathology during 1.5–2.0 months up 0.15 A_450_ units. Three-month-old Th mice showed a low level of anti-DNA Abs in sera (~ 0.03 A_450_) comparable with that for non-autoimmune BALB and CBA mice. However, in contrast to non-autoimmune mice, during 73 days, the concentration of anti-DNA Abs in Th mice increased 3.8- and 5.3-fold (*P* < 0.05) in the case of male and female mice, respectively (Fig. 6a). We evaluated the changes in the concentration of Abs against MBP and MOG over time using homogeneous preparations of IgGs isolated from the sera of male and female mice (Fig. 6c, e). The relative concentration of anti-MBP during 73 days increased for males 1.8-fold, while for female mice, 2.3-fold (*P* < 0.05). As was shown earlier, the blood of C57BL/6 mice, even at 3 months of age, contains MOG and Abs against this antigen [7–10]. A similar situation was revealed for Th mice. The concentration of anti-MOG antibodies in male and female mice at the beginning of the experiment was approximately the same (0.02 A_450_) and increased by about 1.8–2.1 times by day 73 (Fig. 6e). Thus, in contrast to non-autoimmune mice, in Th mice, the concentration of antibodies against DNA, MBP, and MOG increases in time, as in the case of other animals predisposed to the development of spontaneous autoimmune diseases [7–13].

### Time-dependent changes in IgGs catalytic activities

As noted above, the appearance of abzymes with different enzymatic activities is a very important marker of disease onset and the following development for various AIDs [7–13, 25–30]. Non-autoimmune BALB and CBA mice, as well as healthy humans, usually do not demonstrate catalytic activities of antibodies [7–13, 25–30]. The blood of SLE prone MRL-lpr/lpr mice contains abzymes hydrolyzing DNA, ATP, and oligosaccharides [11–13]. EAE prone C57BL/6 mice demonstrated a gradual and nearly linear 6.8-fold increase in DNase activity during spontaneous development of the pathology [7–10].

At time zero, DNase activity of IgGs in Th male mice was ~ 2.5-fold lower than that for female mice (Fig. 6b). However, during 73 days, DNase activity of male IgGs increase 11-fold, while for females, Abs only 2.8-fold (*P* < 0.05) (Fig. 6b). The initial relative activity of IgGs in the hydrolysis of MBP in male and female mice was almost the same (Fig. 6d). However, there was a strong slowdown in the growth of abzyme MBP-hydrolyzing activity in sera of males compared that for females (Fig. 6d). In addition, in time, characters of the changes in the relative concentrations of Abs against DNA and MBP in male and female mice did not coincide with the characters of the change in abzymes activity hydrolyzing these substrates. However, the curves in time changes of the relative concentrations of antibodies against MOG and dependencies for changes in the relative activity of IgGs in MOG-hydrolysis were, to some degree, consistent (Fig. 6e, f). In both cases, a nearly parallel increase in IgGs concentrations and their relative activity in the MOG hydrolysis were observed.

Thus, some noticeable differences are observed for male and female mice not only in the overtime dependencies of changes in differentiation profiles of stem cell (Fig. 1), patterns of changes in lymphocyte proliferation in different organs (Figs. 2–4), but also in relative concentrations of Abs against DNA, MBP, and MOG, as well as IgG activities in their hydrolysis (Fig. 6).

## Discussion

Different AI pathologies was shown to arise after self-tolerance breakdown (central or peripheral) via multiple mechanisms SLE-prone MRL-lpr/lpr, and C57BL/6 mice are predisposed to the spontaneous development of AIDs. The spontaneous development of SLE in MRL-lpr/lpr mice and EAE in C57BL/6 mice may be significantly accelerated by their treatment with DNA and MOG, respectively. Interestingly development of SLE in MRL-lpr/lpr and EAE in C57BL/6 mice leads to very similar changes in the differentiation profile of HSCs and the onset of different abzymes production [7–10].

According to literature data in the case of mammals, both activated myelin-reactive T and B lymphocytes are important for MS pathogenesis [1–3]. Therefore, in this paper, it was interesting to analyze the change in various parameters during the spontaneous development of EAE in Th mice, which are characterized by a T-cell response. In addition, it is known that women are more often affected by multiple sclerosis. Taking this into account, it was interesting to compare the characters of changes in various parameters during the spontaneous development of EAE in males and females of the Th line of mice.

The main difference in time changing the differentiation profiles of bone marrow stem cells for male and female mice was observed for CFU-E (Fig. 1b) and CFU-GEMM cells (Fig. 1d). In addition, when the relative number of B cells in the bone marrow of females and males in time was decreased in a similar way (Fig. 1e), the number of T cells in males decreased, while in females, on the contrary, increased (Fig. 1f).

Interestingly, the relative number of B lymphocytes in the blood, thymus, spleen, and lymph nodes of females and males at 3 months of age was nearly the same or at least comparable (Fig. 2). The character of the changes in their relative amount over time in each individual organ was specific, but there were approximately comparable changes of B lymphocytes in the organs of female and male mice (Fig. 2).

It should be noted that all organs of males and females contain different numbers of T cells (Figs. 3, 4). Interestingly, the relative number of CD4 and Cd8 cells in the bone marrow and blood of male and female mice nearly the same (Figs. 3, 4). In the remaining organs (thymus, spleen, and lymph nodes), the number of CD8 is much less than CD4 cells. However, the nature of the profiles changes of CD8 and CD4 cells in various organs of males and females are very similar to that for changes in total lymphocytes (Figs. 3, 4). Thus, in the process of EAE, spontaneous development in female and male mice demonstrate noticeable differences in bone marrow stem cell differentiation profiles and changes in lymphocyte proliferation in different organs (Figs. 1–4). However, in spite of these differences in males and females, these processes in both cases lead to the production of auto-antibodies against DNA, MBP, and MOG and to abzymes hydrolyzing these substrates (Fig. 6). It is important that the course of the curves of changes in concentration of antibodies against DNA, MBP, and MOG and curves characterizing hydrolysis of these substrates in mice do not well coincide (Fig. 6). Interestingly, there is a significant difference in an increase in time of the relative activity of abzymes hydrolyzing DNA and MBP. In the case of males, the activity of abzymes hydrolyzing DNA increases faster in time than in females, and in females activity of IgGs, hydrolyzing MBP grow up quicker than in males (Fig. 6b, d). The curves characterizing the increase in the concentration of Abs against MOG and the enhancement in activity of abzymes hydrolyzing this substrate are different, but in both cases, there is an increase in these parameters in time (Fig. 6e, f).

According to published data, due to the exceptional heterogeneity and extreme diversity of abzymes hydrolyzing different antigens, each stage of AIDs development may be accompanied by synthesis of many different Abs without catalytic activity and abzymes with very different relative catalytic activities hydrolyzing various antigens [25–30]. Therefore, the different types of the time changes of Abs titers against different antigens and relative activities of abzymes hydrolyzing these antigens may be related to the production of very different auto-Abs with and without catalytic activity at different stages of spontaneous EAE development in Th mice.

In this work, the analysis of several important parameters characterizing the development of spontaneous EAE in Th mice with the T-cell response was carried out for the first time. The proliferation of total lymphocytes, as well as CD8 and CD4 cells during the spontaneous development of AI reactions, was first analyzed. It was shown that the above processes in Th mice were associated with an increase in auto-Abs production, including abzymes.

## Conclusion

It is very likely that for the development of deep autoimmune diseases, mammalian lymphocytes with a T- and B-cell response is required. However, data show that EAE prone Th mice with T-cell response similar to EAE prone C57BL/6 mice with T- and B-cell response predisposed to spontaneous development of AI reactions, associated with a change in the differentiation profiles of bone marrow stem cells, the increase in the level of lymphocyte proliferation in various organs, and the production of harmful for mammals auto-antibodies against different antigens and abzymes that hydrolyze these antigens. It is interesting that the development of EAE in male and female mice of the Th line proceeds to some extent in different ways. In the future, we plan to carry out a more detailed analysis of the mechanisms of EAE development using a comparison of the various parameters for Th mice with T cell response with those for mice with B cell response, and for a hybrid mouse strain with T- and B-cell responses obtained by crossing these two lines.

## Abbreviations

Abs: Antibodies
auto-Abs: Autoantibodies
AI: Autoimmune
AIDs: Autoimmune diseases
BFU-E: Erythroid burst-forming unit (early erythroid colonies)
CNS: Central nervous system
CSF: Cerebrospinal fluid
CFU-GM: Granulocyte–macrophage colony-forming unit CFU-E, erythroid burst-forming unit (late erythroid colonies)
CBA: (CBAxC57BL)F1 mice
CFU-GEMM: Granulocyte-erythroid-megakaryocytic-macrophage colony-forming unit
EPO: Erythropoietin
EAE: Experimental autoimmune encephalomyelitis
HSCs: Hematopoietic stem cells
IL: Interleukin
IgG: Immunoglobulin G
MS: Multiple sclerosis
MTT: Tetrazolium dye MTT 3-(4,5-methylthiazol-2-yl)-2,5-diphenyltetrazolium bromide
MBP: Myelin basic protein
MOG: Myelin oligodendrocyte glycoprotein
SDS-PAGE: Sodium dodecyl sulfate-polyacrylamide gel electrophoresis
sc: Supercoiled
scDNA: Supercoiled DNA
SLE: Systemic lupus erythematosus
